# Effect of Perceived Intimacy on Social Decision-Making in Patients with Schizophrenia

**DOI:** 10.3389/fnhum.2014.00945

**Published:** 2014-11-24

**Authors:** Sunyoung Park, Jung Eun Shin, Kiwan Han, Yu-Bin Shin, Jae-Jin Kim

**Affiliations:** ^1^Department of Psychiatry, Yonsei University College of Medicine, Seoul, South Korea; ^2^Institute of Behavioral Science in Medicine, Yonsei University College of Medicine, Seoul, South Korea; ^3^Severance Biomedical Science Institute, Yonsei University College of Medicine, Seoul, South Korea

**Keywords:** schizophrenia, intimacy, social decision-making, virtual reality

## Abstract

Social dysfunctions including emotional perception and social decision-making are common in patients with schizophrenia. The aim of this study was to determine the level of intimacy formation and the effect of intimacy on social decision in patients with schizophrenia using virtual reality tasks, which simulate complicated social situations. Twenty-seven patients with schizophrenia and 30 healthy controls performed the 2 virtual social tasks: the intimacy task and the social decision task. The first one was to estimate repeatedly how intimate participants felt with each avatar after listening to what avatars said. The second one was to decide whether or not participants accepted the requests of easy, medium, or hard difficulty by the intimate or distant avatars. During the intimacy task, the intimacy rating scores for intimate avatars were not significantly different between groups, but those for distant avatars were significantly higher in patients than in controls. During the social decision task, the difference in the acceptance rate between intimate and distant avatars was significantly smaller in patients than in controls. In detail, a significant group difference in the acceptance rate was found only for the hard requests, but not for the easy and medium difficulty requests. These results suggest that patients with schizophrenia have a deficit in emotional perception and social decision-making. Various factors such as a peculiarity of emotional deficits, motivational deficits, concreteness, and paranoid tendency may contribute to these abnormalities.

## Introduction

Schizophrenia involves a wide range of cognitive, emotional, and behavioral dysfunctions, and no single symptom is pathognomonic of the disorder (American Psychiatric Association, 2000). Among others, social dysfunction has been outweighed as a defining feature for the course of schizophrenia (Olfson et al., 2011). Social dysfunction of patients with schizophrenia mostly results from impaired social cognition (Corrigan and Penn, 2001; Mancuso et al., 2011), which involves deficits in the social relationship. In particular, because recognizing and understanding the others’ thinking and intention may be an important element of building the social relationship, impairments in theory of mind or metacognition may mainly contribute to social dysfunction in patients with schizophrenia (Dimaggio et al., 2008). It may be evidence of this contribution that remediation of impaired social cognition and theory of mind helps improve social functioning in patients with schizophrenia (Combs et al., 2007; Lysaker and Dimaggio, 2014). In addition, impairments in emotional processing (Edwards et al., 2002; Schneider et al., 2006; Butler et al., 2009) and interaction with impaired emotional recognition and theory of mind (Brüne, 2005) need to be considered.

Intimacy may be one of the important factors regarding the social relationship. Intimacy refers to the feeling of being in a close personal association and belonging together. It is a familiar and very close affective connection with others as a result of a bond that is formed through knowledge and experience of them (Laurenceau et al., 1998). Patients with schizophrenia have a difficulty in forming social bonds due to impaired capacity to understand the emotions of others and to express their own emotions (Kulhara et al., 1989; Green et al., 2005). Furthermore, their inability to be attuned to the context of social interactions may lead to social withdrawal and social disability (Salvatore et al., 2007). It should be noted that the ability to form intact intimacy and a desire for intimacy are also necessary for the appropriate intimate relationship with others (Baumeister and Leary, 1995). It has been reported that motivation to intimacy is decreased in patients with schizophrenia due to their symptoms (Hien et al., 1998). It is unclear, however, how much of an impairment they have in intimacy formation toward strangers. Therefore, a focus of the present study was the level of intimacy formation of patients with schizophrenia in experimental situations.

Another focus was a difference in the effect of intimacy on social decision-making between patients with schizophrenia and normal controls. Most decisions related to social situations are dependent on the concomitant choices of others (Sanfey, 2007). Decision-making consists of a complex set of processes, which include reward processing, coordination, and strategic reasoning. Some previous studies have addressed the interactive effect between emotion and decision-making (Hooker and Park, 2002; Tranel et al., 2002; Bechara and Damasio, 2005). In terms of social decision-making, various factors such as competition, social reward, theory of mind, and affection have been proposed to affect this function (Park et al., 1991; Sanfey et al., 2003; Paulus, 2007; Sanfey, 2007). Intimacy may be another example of these factors.

In general, we usually have a tendency to consort with people with positive emotions such as intimate feelings. Conversely, a response to negative emotions like anger reduces cooperation and increases conflict. This pattern of attitude suggests that social decisions are heavily influenced by emotion including intimacy (Van Kleef et al., 2010). It is unclear, however, if the similar feature is also found in patients with schizophrenia who have a deficit in motivation to intimacy. This may be important in providing useful information to a psychosocial rehabilitation program for patients with schizophrenia.

Some factors should be considered in investigation of the effect of intimacy on social decision-making. Because intimacy is an emotion, which is produced in the real social relationship, evaluation of intimacy also needs to be made reflecting complex social situations of real life. This need may be particularly important in patients with schizophrenia who have social dysfunction due to various psychotic symptoms and motivational deficits (Kim et al., 2007). In addition, social anxiety and self-esteem should be taken into account when investigating intimacy levels in patients with schizophrenia (Lysaker et al., 2010). Given that people with social anxiety are used to avoiding risk taking decision (Maner et al., 2007) and those with low self-esteem tend to make decision depending on group-norm rather than their own will (Crocker and Major, 1989; Anthony et al., 2007), social anxiety and self-esteem may be considered to be other factors influencing the effect of intimacy on social decision-making in patients with schizophrenia. In fact, there is evidence that patients with schizophrenia have a high level of social anxiety and low self-esteem, which are closely linked to each other (Karatzias et al., 2007; Lysaker et al., 2008).

In this study, we produced virtual reality tasks, which are suitable for designing controlled, complex social situations to evaluate the behavior of patients with schizophrenia (Park et al., 2011; Han et al., 2012). Virtual reality is a useful technique to simulate various social situations by providing an immersive environment with three-dimensional rendering and a safe experimental environment without the limitation of time and space (Han et al., 2009, 2012). Based on the benefit of virtual reality that emotional and social stimuli are provided in a natural manner and the objective behavioral parameters such as interpersonal distance, reaction time and types of responses are automatically obtained, several studies have performed the estimation of human behaviors in established social situations (Park et al., 2009; Han et al., 2012; Kane et al., 2012).

In the present study, virtual reality was used to construct avatars with whom participants interacted and built intimacy in the complex, dynamic social situations. The purpose of this study was to determine the level of intimacy formation and the effect of intimacy on social decision in patients with schizophrenia. For this purpose, participants’ tasks were to experimentally construct intimacy for avatars and to decide on whether or not to accept the avatar’s request, and the results were compared between patients with schizophrenia and healthy controls. The hypothesis was that (1) patients would have a difficulty in the formation of intimacy with avatars and (2) less intimate avatar’s requests would be rejected in similar proportion between patients and controls, but more intimate avatars’ requests would be less accepted in patients because they might feel less intimate with avatars compared to controls.

## Materials and Methods

### Participants

Twenty-seven patients with DSM-IV-TR (Diagnostic and Statistical Manual of Mental Disorders-IV-Text Revision) (American Psychiatric Association, 2000) schizophrenia were recruited from an outpatient clinic. All patients were medicated with one or two atypical antipsychotics and were clinically stable. Mean illness duration was 9.7 (SD = 4.6) years, and mean Positive and Negative Syndrome Scale (PANSS) (Kay et al., 1987) score for measuring symptom severity was 64.9 (SD = 13.5) (Table 1). Thirty non-psychiatric healthy controls were age- and gender-matched to patients, and were confirmed to have no history of any psychiatric or neurologic illness as diagnosed by a psychiatrist. All participants were aged 28–39 years. Years of education [patients: 14.6 (SD = 2.3), controls: 15.7 (SD = 1.4), *t* = 2.14, *p* = 0.04] were significantly different between the two groups. Considering that social anxiety is associated with avoidant decision-making (Maner et al., 2007), the Liebowitz Social Anxiety Scale (Heimberg et al., 1999) was additionally administered, but did not show a significant group difference. The Rosenberg Self-esteem Scale (Robins et al., 2001) was administered to assess self-esteem effects on social decision-making, and the scores were significantly lower in patients than in controls (*t* = 2.72, df = 45.60, *p* = 0.02). This study was approved by the local institutional review board, and written informed consent was obtained from all participants.

**Table 1:** **Participant characteristics**.

	Control	Schizophrenia	*t*/*x*^2^	*p* value
Age	31.7 (2.1)	33.0 (3.7)	−1.64	0.11
Gender (M:F)	13:17	13:14	0.13	0.72
Education years	15.7 (1.4)	14.6 (2.3)	2.14	0.04
LSAS	39.6 (19.7)	47.1 (29.8)	−1.13	0.27
RSES	30.3 (3.9)	26.8 (5.6)	2.72	0.01
Number of subtypes		Paranoid: 18		
		Undifferentiated: 4		
		Residual: 5		
Illness duration (years)		9.7 (4.6)		
PANSS positive		14.3 (4.4)		
Negative		17.8 (3.8)		
General		32.8 (7.5)		
Total		64.9 (13.5)		

*LSAS, Libowits Social Anxiety Scale; RSES, Rosenberg Self-esteem Scale; SPM, Standard Progressive Matrices, PANSS, Positive and Negative Syndrome Scale. Data are presented as mean (SD)*.

### Design and procedure

All participants were tested with two experimental behavioral tasks, which included several typical everyday environments and four avatar types using virtual reality. The virtual environment was introduced as usual situations in community of participants, and avatars were supposed to be acquaintances. The experimental tasks were constructed using Game Studio A6 Engine (Conitec Datasystems, oP Group).

#### Intimacy task

**Figure 1:**
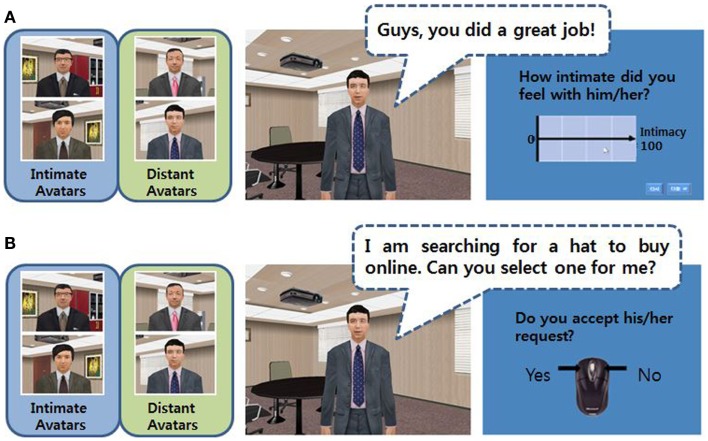
**The virtual intimacy (A) and social decision (B) tasks**.

This task aimed to build intimacy with virtual avatars before the next task was administered, and to estimate the level of intimacy that participants feel with four avatars. Two avatars were constructed to be intimate (referred to as “intimate avatars”), and the others were constructed to be distant (referred to as “distant avatars”). Avatars spoke five times to participants in a manner of familiar and informal relationship (e.g., the weather was really good last Sunday! Did you go outside with your family?) or unfamiliar and formal relationship (e.g., we are not going to make progress any more. Let’s take a rest.), respectively. Appearance, voice tone, and politeness in manner of speech were carefully constructed to reflect their assigned intimacy level. After listening to what avatars said, participants used a mouse to estimate how intimate they felt with each avatar using a Likert scale, which ranged from 0 to 100 and was marked at every 25 points (Figure 1A). Participants were instructed to rate above 50 if felt intimate and below 50 if felt distant. The intimacy score for each avatar type was defined as a mean score for 10 trials for two intimate or two distant avatars. There was no time limit for this task. In the preliminary validity test in 20 normal volunteers who did not participate in the main experiment, intimate avatars were rated above 55 and distant avatars were rated below 45.

#### Social decision task

This task was constructed to assess participants’ social decision-making in complicated social situations. Avatars, which participants had become familiar with during the intimacy task, requested them to do something. The requests were made at three levels of difficulty: easy (e.g., I am searching for a hat to buy online. Can you select one for me?), medium (e.g., I have left my cell phone at home. Can I use your cell phone for an hour?), and hard (e.g., I need to go on a trip with my friends next week. May I use your car next week?). The dialogues in the tasks were reported in Supplementary Material. They were selected from the requests classified as one of the three categories in the preliminary validity test. The category was made according to the responses of 20 normal volunteers who also participated in the preliminary validity test for the intimacy task: “easy” if accepted by above 60%, “medium” if accepted between 40 and 60%, and “hard” if accepted only below 40%. Four avatars made 9 easy, 9 medium, and 9 hard requests, and thus 108 trials were included in the task. Participants were asked to decide whether or not they would accept the request by clicking a corresponding mouse button (Figure 1B). Participants were asked to make the decision as quickly as possible before the next trial began. The asking period was 5 s for all requests, and the following responding period persisted for 4 s. Scores were 1 for acceptance and 0 for refusal, and average scores for each avatar were considered to be the acceptance rate. In addition, reaction time was automatically counted as the time between the start of the responding period and the clicking response. Reaction times of acceptance or refusal responses were merged and analyzed together.

### Statistical analysis

Demographic characteristics were compared between groups using the Student’s *t*-test and Chi-square test. Considering that our data included several missing responses, especially in patients, the behavioral performances such as the intimacy score, acceptance rate, and reaction time were analyzed using a mixed linear model. The variables for the main and interaction effects were *avatar type* (intimate avatars and distant avatars) and *group* (patients and controls) for the intimacy score, and were *avatar type*, *request difficulty* (easy, medium and hard), and *group* for the acceptance rate and reaction time. Considering the missing data, LSMEANS was used to report behavioral results. Pearson correlations of the behavioral performances with scores on the Liebowitz Social Anxiety Scale and Rosenberg Self-esteem Scale in each group and PANSS scores in patients were calculated. When analyzing behavioral data, years of education were used as a covariate.

## Results

### Intimacy rating

**Figure 2:**
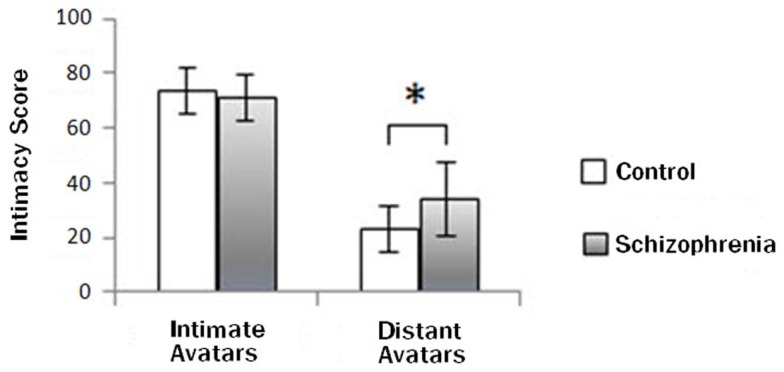
**Group difference in the intimacy rating scores**. The intimacy rating scores for distant avatars were significantly higher in patients than in controls (*p < 0.01), but not for intimate avatars.

The intimacy rating scores had the significant main effect of avatar type (Num DF = 1, Den DF = 169, *F* = 1,057.04, *p* < 0.01) and group (Num DF = 1, Den DF = 169, *F* = 4.49, *p* = 0.04). They were significantly higher for intimate avatars (LSMEANS: 72.4 ± 1.20) than for distant avatars (LSMEANS: 28.5 ± 1.2), and were significantly higher in patients (LSMEANS: 52.5 ± 1.4) than in controls (LSMEANS: 48.4 ± 1.3). The intimacy scores showed the significant interaction effect between avatar type and group (Num DF = 1, Den DF = 169, *F* = 25.71, *p* < 0.01). As shown in Figure 2, the intimacy scores for intimate avatars were not significantly different between groups (LSMEANS: patients, 71.0 ± 1.7; controls, 73.7 ± 1.6; *p* > 0.05), but those for distant avatars were significantly higher in patients than in controls (LSMEANS: patients, 34.0 ± 1.7; controls, 23.0 ± 1.6; *p* < 0.01).

### Acceptance rate

As shown in Table 2, the significant main effect was found in avatar type; the acceptance rates were higher for intimate avatars than for distant avatars (Num DF = 1, Den DF = 617, *F* = 71.42, *p* < 0.01). The significant main effect of request difficulty was also revealed (Num DF = 2, Den DF = 617, *F* = 275.13, *p* < 0.01); *post hoc* analysis demonstrated that the acceptance rates were significantly different between the request difficulty levels (easy > medium, *p* < 0.01; medium > hard, *p* < 0.01).

**Table 2:** **LSMEANS (SD) of the acceptance rate and reaction time according to avatar type, request difficulty, and group**.

			Control	Schizophrenia	*p* value
Acceptance rate (0–1)	Avatar type	Intimate	0.63 (0.15)	0.54 (0.16)	0.07
		Distant	0.42 (0.13)	0.44 (0.15)	0.93
	Request difficulty	Easy	0.76 (0.16)	0.80 (0.14)	0.94
		Medium	0.49 (0.15)	0.48 (0.20)	0.99
		Hard	0.31 (0.14)	0.19 (0.18)	0.03
Reaction time (s)	Avatar type	Intimate	0.82 (0.20)	0.98 (0.29)	0.08
		Distant	0.77 (0.23)	0.90 (0.27)	0.07
	Request difficulty	Easy	0.72 (0.23)	0.92 (0.30)	0.02
		Medium	0.82 (0.23)	0.99 (0.30)	0.06
		Hard	0.84 (0.22)	0.92 (0.27)	0.57

**Figure 3:**
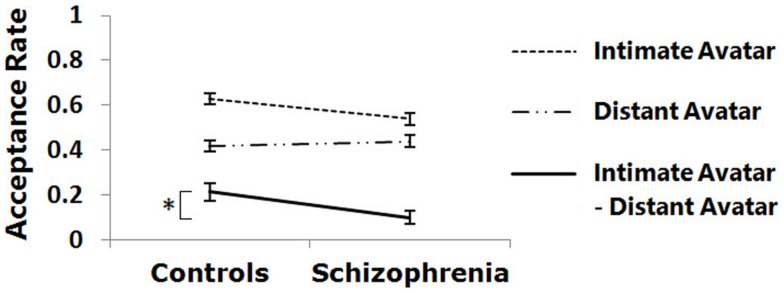
**Group difference in the acceptance rate is shown**. Increases of the acceptance rate for intimate avatars versus distant avatars were significantly smaller in patients than in controls (*p < 0.05).

There was no main effect of group, but the interaction effect was found between avatar type and group (Num DF = 1, Den DF = 617, *F* = 9.40, *p* < 0.01). As shown in Figure 3, increases of the acceptance rate for intimate avatars compared with distant avatars were significantly smaller in patients than in controls (*p* < 0.05). The significant interaction effect was also found between request difficulty and group (Num DF = 2, Den DF = 617, *F* = 6.66, *p* < 0.01). In both avatar types, a significant group difference was found only for the hard requests (*p* < 0.05), but not for the easy and medium difficulty requests. There was no significant interaction between avatar type, request difficulty, and group.

### Reaction time

As shown in Table 2, the significant main effect of group was found in reaction time, which was longer in patients than in controls (Num DF = 1, Den DF = 617, *F* = 7.12, *p* < 0.01). The significant main effect of avatar type was also found; reaction time was longer for intimate avatars than for distant avatars (Num DF = 1, Den DF = 617, *F* = 19.64, *p* < 0.01). The main effect of request difficulty was also significant (Num DF = 2, Den DF = 617, *F* = 10.21, *p* < 0.01); reaction time was significantly shorter to the easy requests than to the medium requests (*p* < 0.01), but there was no difference between the easy and difficult requests and between the medium and difficult requests.

The significant interaction effect was not shown between avatar type and group, but found between request difficulty and group (Num DF = 2, Den DF = 617, *F* = 4.59, *p* = 0.01); in both avatar types, reaction time for the easy requests was significantly longer in patients than in controls (*p* < 0.05), but not for the medium or hard requests. No interaction effect was found between avatar type, request difficulty, and group.

### Correlations

**Figure 4:**
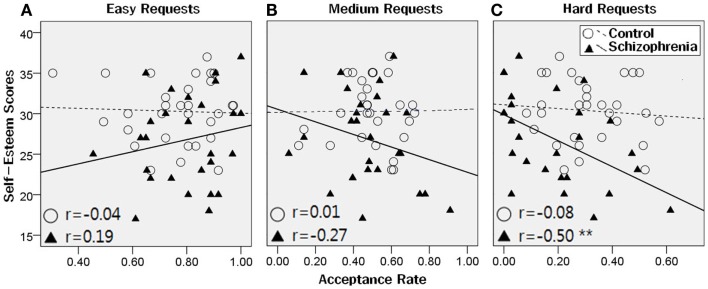
**Correlations between the acceptance rates and the Rosenberg Self-esteem Scale scores**. There were no significant correlations between the Rosenberg Self-esteem Scale scores and the acceptance rates for the easy and medium requests, in both groups **(A, B)**. For the hard requests **(C)**, the Self-esteem scores were significantly correlated with the acceptance rates in patients (**p < 0.001), but not in controls.

The Rosenberg Self-esteem Scale scores were only significantly correlated with the acceptance rates for the hard requests in patients (*r* = −0.50, *p* = 0.007), but not in controls (Figure 4). The Liebowitz Social Anxiety Scale scores were not significantly correlated with the acceptance rates or reaction times for any request difficulty level in both groups. In patients, the PANSS total scores were significantly correlated with reaction time (*r* = 0.57, *p* = 0.002). In detail, the correlation was significant with the negative symptom scores (*r* = 0.54, *p* = 0.004), but not with the positive symptom scores (*r* = 0.37, *p* = 0.06).

## Discussion

In the present study, we examined the difference in the level of intimacy formation and the effect of intimacy on social decision between patients with schizophrenia and normal controls. Both patients and controls tended to feel closer to intimate avatars than distant avatars and to accept requests less often as requests grew more difficult. These results suggest that virtual reality tasks simulating complex social situations of real life can be effectively applied to both groups. As expected, however, compared with controls, patients showed a different pattern in the formation of intimacy with avatars and refused the distant avatars’ requests more often.

### Intimacy rating

The intimacy rating scores for intimate avatars during the intimacy task were not different between patients and controls, but those for distant avatars were higher in patients than in controls, suggesting that there is a difference in social cue-based emotional cognition between the two groups. This is in line with previous findings that patients with schizophrenia have impairment in social cognition, including emotion perception, theory of mind, and attributional style (Penn et al., 2008). In particular, inappropriate intimacy rating for distant avatars in patients may reflect deficits in emotion perception, which were reported to be prominent in schizophrenia (Pinkham et al., 2007).

In general, expressing intimacy is positive and affiliative, whereas expressing distance is awkward and estranged (Feeney, 1999). Attitudes of distant avatars could be perceived as rude or brazen on participants, and these could induce negative emotion. Therefore, our results may reflect a bias of patients with schizophrenia toward negative emotional stimuli, as revealed in several behavioral studies investigating emotional perception (Kinderman, 1994; Loughland et al., 2002; Choi et al., 2010). A previous study using a role-play with various situations demonstrated that patients with schizophrenia consistently underestimated the intensity of negative emotion, but not the intensity of positive emotion (Bellack et al., 1992). This pattern of behaviors might be an expression of denial to the aversive condition or a coping response to control for the overwhelming and aversive input. Taken together, the feature of feeling more intimate with distant avatars rather than feeling more distant with intimate avatars reflects a peculiarity of emotional deficits in schizophrenia.

Alternatively, abnormal theory of mind might contribute to the findings of impaired intimacy formation. In other words, it could have been difficult for patients with impaired theory of mind to understand intentions of avatars in various situations, which were included in the intimacy task. This possibility is consistent with a previous report that accurate mind-reading was important to building an intimate relationship (Thomas and Fletcher, 2003).

### Acceptance rate

Patients showed no difference compared with controls in the acceptance rate for each of intimate and distant avatars during the social decision task, suggesting that patients with schizophrenia have the ability to take intimacy into account similarly to control subjects while accepting or rejecting the requests. However, further analyses showed that the difference in the acceptance rate between intimate and distant avatars was smaller in patients than in controls, suggesting that the effect of intimacy on social decision seems to be relatively small in schizophrenia.

Furthermore, during the social decision task, patients showed similar acceptance rate for the easy and medium difficulty requests when compared with controls, but lower acceptance rate for the hard requests than controls, suggesting motivation deficits in schizophrenia. Although patients with schizophrenia are able to predict and value social factors such as reciprocity and equity, the ability to feel anticipatory pleasure for social reward may be insufficient to motivate them to incur greater cost (Choi et al., 2013). Therefore, our findings during the social decision task can be interpreted as an aspect of negative symptoms in a broad sense (Gorissen et al., 2005; Choi et al., 2013).

Alternatively, concreteness of patients may have an effect on the results. When a represented situation becomes complex, individuals may need abstract thinking or flexibility to consider various factors, including familiarity, reciprocity, equity, and hierarchy. Given that patients with schizophrenia are known to be more impaired when making more abstract social judgments (Penn et al., 1997), the difference in the hard requests can be explained as being attributed to the patients’ concrete judgment in relation to rising situational complexity. Another possibility of lower acceptance rate for the hard requests in patients could be attributed to their paranoid tendency. In the current study, 66.7% of patients had the paranoid subtype. As a request gets harder, paranoid patients can interpret it as a threatening or exploiting one.

### Effects of self-esteem and social anxiety

Meanwhile, patients with stronger self-esteem showed a tendency to refuse the hard requests more often. Our study was conducted in an Asian country in which collectivistic value orientation is dominant (Jackson et al., 2006; Dierdorff et al., 2011). In this cultural background, it is typical for people to take count of the personal relationship, and thus healthy controls might have accepted the hard requests regardless of their self-esteem. However, patients with schizophrenia who have deficits in social and emotional functioning might have been more influenced by self-esteem. More independent and less interdependent features are shown to predict higher self-esteem levels (Singelis et al., 1999).

We expected similar influence by social anxiety, but it did not produce any positive result. Given that a strong correlation between self-esteem and social anxiety has been reported in patients with schizophrenia (Lysaker et al., 2008), this negative result is somewhat of a surprise. This result may reflect a characteristic of our participants, who showed a significant group difference in self-esteem, but not in social anxiety. If patients with severe social anxiety had been more recruited, the result could have been changed.

### Reaction time and symptom severity

The results of overall reaction time appeared to be very short, probably because the presenting time of requests was sufficiently long that participants would have already made the decision even before the start of the separately given responding time. Nonetheless, our results showed that patients reacted more slowly, and as their symptom severity increased, reaction time also increased. This feature may correspond to previous studies, which revealed significant relationships between symptoms, cognitive impairment, and reaction time (Smyrnis et al., 2009; Neill and Rossell, 2013). In particular, the significant correlation of delayed reaction time was found with the negative symptom scores, but not with the positive symptom scores. A previous study reported that negative symptoms were correlated with the simple reaction time tasks in patients with persistent illness rather than patients with fluctuating illness, suggesting that persistent illness, negative symptoms, and impaired initiation may reflect enduring brain structural abnormalities (Ngan and Liddle, 2000). Therefore, it needs to be considered that delayed reaction time in patients may be related to the severity of negative symptoms and poor outcomes.

### Clinical implication and limitations

Our findings provide additional information for clinical practitioners. Various therapies are available, such as social skills training to help patients with schizophrenia cope with the affiliative relationships and roles required for independent living (Hogarty et al., 1986; Kopelowicz et al., 2006). To build up a patient’s social repertoire to a proficient level, therapists must train a wide skill spectrum, which includes social perception, social information processing, affiliative skills, and so on (Kopelowicz et al., 2006). In this study, patients showed a difficulty in the intimacy formation and inappropriate acceptance for the requests. During therapy, if understanding of various social cues and social reward expectations can be enhanced, clinicians can lead patients to be more affiliative. In particular, a training program using virtual reality can be especially beneficial in improving motivation to participate in the therapy and enhancing conversational and assertiveness skills (Park et al., 2011).

There were some limitations in the present study. The level of education was significantly lower in patients than in controls, and thus it was included as a covariate. Comprehensive cognitive measures were not applied, and thus we did not know if our findings of social deficits were related to cognitive dysfunctions. A small sample size was another limitation. Larger sample size would produce more different levels of emotions on social behaviors. In addition, there was some task weakness with respect to enhancing participants’ intimacy with avatars. In a previous study, interactive situations were shown to be necessary for participants to feel intimate with avatars and situations (Kane et al., 2012). In the present study, however, participants did not have a chance to interact with avatars, and this could have prevented participants from feeling like experiencing a real social relationship. It was also a limitation that the feeling of reality during the tasks was not evaluated using an appropriate scale.

## Conclusion

The present study for determining the level of intimacy formation and the effect of intimacy on social decision using virtual reality tasks revealed that patients with schizophrenia showed significantly higher intimacy rating scores for distant avatars during the intimacy task than controls, and significantly smaller increases of the acceptance rate for intimate avatars compared with distant avatars during the social decision task than controls. In addition, patients tended to refuse more the hard requests than controls. These results suggest that patients with schizophrenia have a deficit in emotional perception and social decision-making. Various factors such as a peculiarity of emotional deficits, motivational deficits, concreteness, and paranoid tendency may contribute to these abnormalities. Our results provide additional information on impairments in social cognition and social interaction in patients with schizophrenia.

## Conflict of Interest Statement

The authors declare that the research was conducted in the absence of any commercial or financial relationships that could be construed as a potential conflict of interest.

## Supplementary Material

The Supplementary Material for this article can be found online at http://www.frontiersin.org/Journal/10.3389/fnhum.2014.00945/abstract
