# Supplementary material for: Effect of Perceived Intimacy on Social Decision-Making in Patients with Schizophrenia
Source: Front Hum Neurosci. 2014 Nov 24;8:945. doi: 10.3389/fnhum.2014.00945 (PMC4241746; doi:10.3389/fnhum.2014.00945)
Supplement: Supplementary file 1 [file Table_1.DOCX]

**SUPPLEMENTARY MATERIAL**

INTIMACY TASK: Dialogue

***Intimate avatar 1.***

1. The weather was really good last Sunday! Did you go outside with your family?
2. Are you busy now? I’m going to have a cup of coffee, would you like to join me?
3. Your presentation today was really good! I have nobody but you to rely on.
4. Do you have any problem? You look so tired.
5. I know that you have been doing well at conferences. Please help me prepare some documents for a conference.

***Intimate Avatar 2.***

1. It was a really good idea! Would you share some of yours with me?
2. Did you really do these things alone? I can’t believe it. You did a great job.
3. I’m so tired. I think that I can do better if you buy me a cup of coffee.
4. I heard you had so much work to do last week. Did you take a rest during the weekend?
5. Oops, you’ve sent me wrong data. I should do it all again. You should take the consequences (kiddingly).

***Distant Avatar 1.***

1. We are not going to make progress any more. Let’s take a break.
2. There are so much missing data in this paper. Complete it and report again.
3. Well done. Keep going.
4. Did you spend a good weekend?
5. Your presentation was impressive.

***Distant Avatar 2.***

1. Hello, sir. Did you spend a good weekend?
2. If you send me the data, I will deal with it.
3. Yes, sir. I think that’s a good idea.
4. Excuse me, sir. Can I take a break for a while?
5. Something is missing in this paper. If you don’t mind, would you check this again, please?

SOCIAL DECISION TASK: Dialogue

***Easy requests***

1. I am searching for a hat to buy online. Could you select one for me?
2. My PC is out of order. Can I use your laptop for a moment?
3. Could you call the charter bus service and find out the price?
4. I have a lot of luggage to carry, so could you call the taxi for me?
5. Could you take me to the nearest subway station on your way out?
6. I forgot to turn off the PC because I was in a hurry. Could you turn off my computer for me?
7. Time is up. Could you announce the instruction of Sports Day to the attendees?
8. I will travel abroad. Could you check out the car rental information?
9. I can hardly see anything through the window because of the frost over there. Could you wipe the window a bit?

***Medium requests***

1. I have been transferred to another office. Could you move my PC to the next room?
2. I had a good dream yesterday. Could you buy me a lottery ticket from a nearby store?
3. I have left my cell phone at home. Can I use your cell phone for an hour?
4. My watch is out of order. Could you have it repaired in (repair shop in) the department store for me?
5. I want to get a driver’s license. Can you go to the DMV office and make a reservation for a driving test for me?
6. Can you pick up my children from the kindergarten?
7. My PC is slowing down. Could you format my PC and reinstall the Windows?
8. I have broken the fish bowl by my mistake. Could you clean it for me right now?
9. I have made a membership directory of the alumni association. Could you sell it to all the members of the association?

***Hard requests***

1. Could you move the refrigerator upstairs instead of me?
2. I will be moving to a new house this weekend and I will be so busy. Could you move my luggage instead of me this weekend?
3. I really want to have some fresh raw fish. Could you buy one for me from a dining restaurant near Sokcho port early in the morning?
4. I will go on a trip with my friends next week. Can I use your car for next week?
5. I have got into a car accident and it’s my fault. Could you settle the car accident claim for me?
6. I’ve heard there are a lot of car thieves these days. Could you keep an eye on my car through the entire night?
7. My grandfather living in the countryside will visit the city. Could you show him around?
8. My cat has died. Could you bury my cat in the ground for me?
9. My father has been hospitalized. Could you take care of him in the hospital instead of me?
